# Impact of Consuming Extra-Virgin Olive Oil or Nuts within a Mediterranean Diet on DNA Methylation in Peripheral White Blood Cells within the PREDIMED-Navarra Randomized Controlled Trial: A Role for Dietary Lipids

**DOI:** 10.3390/nu10010015

**Published:** 2017-12-23

**Authors:** Ana Arpón, Fermín I. Milagro, Cristina Razquin, Dolores Corella, Ramón Estruch, Montserrat Fitó, Amelia Marti, Miguel A. Martínez-González, Emilio Ros, Jordi Salas-Salvadó, José-Ignacio Riezu-Boj, J. Alfredo Martínez

**Affiliations:** 1 Department of Nutrition, Food Sciences and Physiology, University of Navarra, 31008 Pamplona, Spain; aarpon.1@alumni.unav.es (A.A.); fmilagro@unav.es (F.I.M.); amarti@unav.es (A.M.); jiriezu@unav.es (J.-I.R.-B.); 2 Centre for Nutrition Research, University of Navarra, 31008 Pamplona, Spain; 3 Spanish Biomedical Research Centre in Physiopathology of Obesity and Nutrition (CIBERobn), Institute of Health Carlos III, 28029 Madrid, Spain; crazquin@unav.es (C.R.); dolores.corella@uv.es (D.C.); restruch@clinic.ub.es (R.E.); mfito@imim.es (M.F.); mamartinez@unav.es (M.A.M.-G.); EROS@clinic.ub.es (E.R.); jordi.salas@urv.cat (J.S.-S.); 4 Department of Preventive Medicine and Public Health, University of Navarra, 31008 Pamplona, Spain; 5 Navarra Institute for Health Research (IdiSNa), 31008 Pamplona, Spain; 6 Department of Preventive Medicine and Public Health, University of Valencia, 46010 Valencia, Spain; 7 Department of Internal Medicine, Institut d’Investigacions Biomediques August Pi i Sunyer (IDIBAPS), Hospital Clinic, University of Barcelona, 08036 Barcelona, Spain; 8 Institut de Recerca Hospital del Mar de Investigacions Mèdiques (IMIM), 08003 Barcelona, Spain; 9 Lipid Clinic, Department of Endocrinology and Nutrition, Institut d'Investigacions Biomediques August Pi i Sunyer (IDIBAPS), Hospital Clinic, University of Barcelona, 08036 Barcelona, Spain.; 10 Human Nutrition Department, Hospital Universitari Sant Joan, Institut d’Investigació Sanitaria Pere Virgili, Universitat Rovira i Virgili, 43201 Reus, Spain; 11 Madrid Institute of Advanced Studies (IMDEA), IMDEA Food, 28049 Madrid, Spain

**Keywords:** Mediterranean diet, DNA methylation, nuts, olive oil, blood cells

## Abstract

DNA methylation could be reversible and mouldable by environmental factors, such as dietary exposures. The objective was to analyse whether an intervention with two Mediterranean diets, one rich in extra-virgin olive oil (MedDiet + EVOO) and the other one in nuts (MedDiet + nuts), was influencing the methylation status of peripheral white blood cells (PWBCs) genes. A subset of 36 representative individuals were selected within the PREvención con DIeta MEDiterránea (PREDIMED-Navarra) trial, with three intervention groups in high cardiovascular risk volunteers: MedDiet + EVOO, MedDiet + nuts, and a low-fat control group. Methylation was assessed at baseline and at five-year follow-up. Ingenuity pathway analysis showed routes with differentially methylated CpG sites (CpGs) related to intermediate metabolism, diabetes, inflammation, and signal transduction. Two CpGs were specifically selected: cg01081346–*CPT1B*/*CHKB-CPT1B* and cg17071192–*GNAS/GNASAS*, being associated with intermediate metabolism. Furthermore, cg01081346 was associated with PUFAs intake, showing a role for specific fatty acids on epigenetic modulation. Specific components of MedDiet, particularly nuts and EVOO, were able to induce methylation changes in several PWBCs genes. These changes may have potential benefits in health; especially those changes in genes related to intermediate metabolism, diabetes, inflammation and signal transduction, which may contribute to explain the role of MedDiet and fat quality on health outcomes.

## 1. Introduction

Epigenetics has been defined as the study of heritable changes that cannot be explained through variations in DNA nucleotide sequence [1], but can result in alteration of gene expression, providing a connection among genetics, diseases, and the environment [2]. Actually, epigenetic alterations have been associated to several diseases and complications, such as obesity, type 2 diabetes, adverse cardiovascular events, or immune diseases [3]. Accumulating evidence suggests that epigenetic marks are reversible [2,4] and they can be modulated by environmental factors [5,6]. Interestingly, nutrients and specific dietary components of the diet are able to modify gene expression through changes in DNA methylation [3]. Thus, the study of nutritional biomarkers and the progress in the epigenetics field are contributing not only to define new nutrient roles in health and disease, but also to the implementation of precision nutrition strategies [7].

Mediterranean diet (MedDiet) has been associated with a decrease in the risk of cardiovascular events, with a favourable effect on blood pressure, insulin sensitivity, lipid profile, inflammation, oxidative stress, and metabolic syndrome features [8,9,10]. Interestingly, MedDiet has been involved in changes in DNA methylation. For instance, a preliminary study within the PREvención con DIeta MEDiterránea (PREDIMED) trial reported associations between MedDiet and methylation at 1-year in *FTO* (Alpha-ketoglutarate dependent dioxygenase) and *TCF7L2* (Transcription factor 7 like 2) genes [11]. Another ancillary study within PREDIMED-Navarra study described an association between adherence to MedDiet and methylation of several inflammation-related genes in peripheral white blood cells [12].

Although extra-virgin olive oil (EVOO) and nuts have been previously related to DNA methylation changes [13,14], no new relationships between the different MedDiets (enriched in either EVOO or nuts) from the PREDIMED trial and methylation have been described before. Therefore, the aim of the current investigation was to explore methylation changes, which were caused by two diets with Mediterranean profiles, in genes of peripheral white blood cells (PWBCs) from participants in the PREDIMED-Navarra trial with emphasis on the impact of fat-quality.

## 2. Materials and Methods

### 2.1. Study Design and Participants

The current study was conducted within the framework of the PREDIMED trial. Briefly, PREDIMED was a multicentre, randomized, primary prevention feeding trial with blinded assessment of end points carried out in Spain with the aim of evaluating the effects of the MedDiet on primary cardiovascular prevention (www.predimed.es). The study design has been described elsewhere [15,16], and it was approved by the Research Ethics Committees at all of the recruiting centres in compliance with the Helsinki Declaration. The Institutional Review Board of the Navarra recruitment centre approved the study protocol (protocol 50/2005). All participants provided written informed consent. This trial was registered with the International Standard Randomised Controlled Trial Number system 35739639.

Eligible participants in PREDIMED trial were men aged 55–80 and women aged 60–80 years without any previous history of cardiovascular disease. Inclusion and exclusion criteria have been described elsewhere [15,16]. Participants were randomized to one of three nutrition interventions: a MedDiet supplemented with EVOO (MedDiet + EVOO), a MedDiet supplemented with mixed nuts (MedDiet + nuts), or advice in reducing all sources of fat, which was the control group. All of the groups received dietary training and questionnaires about medical conditions, food consumption (such as 137-item validated food-frequency questionnaire) [17], and physical activity (Minnesota Leisure-Time Physical Activity Questionnaire) [16] were completed as described elsewhere [15]. Data of anthropometric measures, body composition and blood pressure were collected in the same consultations following standardized procedures [10]. Plasma, serum, and buffy-coat were stored (−80 °C) and biochemical features were evaluated, as described elsewhere [10].

For this study, participants were selected from the recruitment centre at the University of Navarra following different criteria: non-smokers or former-smokers, same proportion of women and men, and aged between 60 and 70 years old, enrolling a total of 36 participants (12 from each diet) with data at baseline and at five years of intervention.

### 2.2. DNA Extraction and DNA Methylation Analysis

The procedures for DNA extraction from buffy coat (PWBCs) of venous blood samples have been described elsewhere [12]. The extracted DNA was sent on dry ice to Unidad de Genotipado y Diagnóstico Genético from Fundación Investigación Clínico de Valencia, where bisulphite treatment was performed and Infinium HumanMethylation450K bead chip (Illumina, San Diego, CA, USA) was used for DNA methylation analysis as described elsewhere [12].

### 2.3. Treatment of Methylation Raw Data

Normalization of microarray data was performed in R by a categorical subset quantile normalization method using the pipeline developed by Touleimat & Tost [18]. After data normalization, Linear Models for Microarray Data (LIMMA) [19] was used to identify the probes with significant differential methylation changes (five years–baseline) between two diets, being the comparisons: MedDiet + EVOO vs. low-fat control diet and MedDiet + nuts vs. low-fat control diet. The linear model was adjusted for age, sex, and microarray chip. The data discussed in this publication have been deposited in NCBI’s Gene Expression Omnibus [20] and are accessible through GEO Series accession number GSE107205 (https://www.ncbi.nlm.nih.gov/geo/query/acc.cgi?acc=GSE107205).

### 2.4. Ingenuity Pathway Analysis

From all the significant CpGs that were obtained after LIMMA analysis, a selection was carried out in order to have an appropriate number of CpGs for obtaining relevant results in the analysis of canonical pathways by Ingenuity Pathway Analysis (IPA) platform (Qiagen Redwood City, CA, USA, www.ingenuity.com). CpGs with a *p* < 0.005 and effect size ≤−9 or ≥9 (the effect size represents the methylation change for the CpG site in %) were selected. Predefined pathways and functional categories of the Ingenuity Knowledge Base were used in order to detect associated pathways and relevant gene regulatory networks [21]. Canonical pathways with a *p* < 0.05 after Fisher’s test were defined as a statistically significant overrepresentation of input genes in a given process.

### 2.5. Differential CpG Site Selection

In order to select CpGs presenting a higher effect on methylation with biological implications that could be noticeable and minimize the type I error rate, a series of statistically restricted selections with stringent criteria were performed. CpG sites were selected by B-values (statistic of differential methylation generated in LIMMA package) >0 and effect size ≤−9 or ≥9 for both of the comparisons. Moreover, methylation values must not be significantly different among the three dietary groups at baseline, but show differences throughout the intervention. The tests that were used in these cases were ANOVA with Tukey’s multiple comparison test or Kruskal-Wallis with Mann-Whitney’s U test, when appropriate, and Student-*t* test or sign test, when appropriate. Then, regression models between selected CpGs methylation and foods or their components, adjusted for diabetes and BMI, were performed.

### 2.6. Blood Cell Type Composition

Differences in the composition of types of leukocytes between five years and baseline were correlated (Pearson) with nuts intake at five years, EVOO consumption at five years and with methylation of the CpGs selected. Differences among dietary groups in the composition of blood cells were also assessed.

### 2.7. Statistical Analysis of Participants

Anthropometric and biochemical features of participants and food consumption were characterised and tested comparing the three groups at baseline, at five years and their differences (five years-baseline) using ANOVA or Kruskal-Wallis test (for quantitative variables) and Chi-square test, Fisher’s exact test, or Cochran test (for qualitative variables), as appropriate. The values were also compared for each diet between five years and baseline using Student *t*-test, Wilcoxon signed-rank test, Sign test, or Cochran test, when advised. The Shapiro-Wilk analysis was used to test normality.

Statistical calculations and graphs were performed using STATA version 12.0 (Stata Corp, College Station, TX, USA) and GraphPad Prism 6 (Graph-Pad Software, San Diego, CA, USA), respectively. The statistically significant level was set at *p* < 0.05.

## 3. Results

### 3.1. Participants’ Differences in Anthropometric and Biochemical Features, Food Consumption and Blood Cell Type Composition

The three dietary groups showed no differences in anthropometric and biochemical features, except for HDL cholesterol levels measured at five years. Concerning clinical manifestations, the three groups exhibited a significant decrease in diabetes, hypercholesterolemia, and arterial hypertension cases after five years of intervention, except for diabetes in low-fat control diet and for arterial hypertension in MedDiet + nuts group, which did not present significant differences (Table 1).

Energy and nutrient intakes were also recorded (Table 2). At the baseline, no significant differences between groups were noted. The highest significant differences were observed for nuts and EVOO consumption at five years among the three groups. Moreover, EVOO consumption was statistically different between five years and baseline in MedDiet + EVOO group. Some differences were also observed in fruit intake between five years and baseline in low-fat control diet and MedDiet + nuts group, as well as in red meat intake in MedDiet + EVOO group. There were also differences in legumes intake between five years and baseline in MedDiet + nuts group and among the three groups.

Since methylation measured in PWBCs can vary by blood cell type, and thus the methylation changes that are associated with the variables investigated in this study may reflect an alteration in blood cell composition, correlation studies were performed between the relative proportions of granulocytes, lymphocytes, or mid cells and nuts intake, EVOO intake or methylation of the selected CpGs, but no associations for any comparison were found (Supplementary Materials Table S1). There were also no differences in blood cell types among the three dietary groups (Supplementary Materials Table S2).

### 3.2. Relevant Differentially Methylated CpGs Were Related to Metabolism, Inflammation, Intracellular Signals and Diabetes

For IPA analysis, 223 CpGs from MedDiet + EVOO vs. low-fat control diet comparison and 359 CpGs from MedDiet + nuts vs. low-fat control diet were analysed. The obtained canonical pathways were collected for both comparisons (Supplementary Materials Table S3 and Table S4, respectively). Some CpGs from both of the comparisons were related to metabolism, such as Fatty Acid Activation, γ-linolenate Biosynthesis II, Fatty Acid β-oxidation I, Fatty Acid α-oxidation, and Adipogenesis pathway. CpGs from MedDiet + EVOO vs. low-fat control diet comparison were related to pathways that were involved in inflammation, such as Leukocyte extravasation signaling, LPS/IL-1 Mediated Inhibition of retinoid acid receptor function, and Crosstalk between Dendritic Cells and Natural Killer Cells. On the other hand, CpGs from MedDiet + nuts vs. low-fat control diet comparison were related to a variety of intracellular signals (apoptosis and DNA damage, Protein kinase A, Notch, G-Protein coupled receptor, Stress-activated protein kinase/c-Jun NH(2)-terminal kinase, etc.), and with diabetes (Type II Diabetes Mellitus Signalling).

### 3.3. Two Differentially Methylated CpGs (cg01081346 and cg17071192) Were Selected

**Figure 1:**
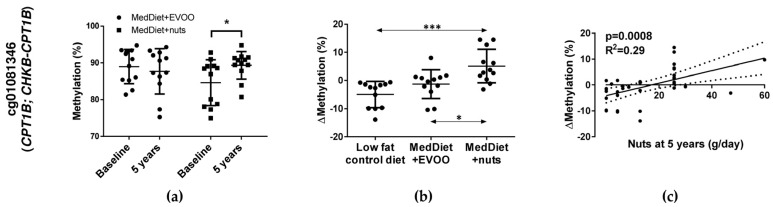
Representation of significant methylation changes of cg01081346. (**a**) Methylation mean and SD of each dietary group at baseline and at 5 years. Statistical analysis was performed by a Student-*t* test between 5 years and baseline for each diet. (**b**) Methylation changes (Mean and SD) for each participant and diet. Statistical analysis of differences in methylation changes among diets was performed by ANOVA (+Tukey’s multiple comparison test). Significance is considered * *p* < 0.05, *** *p* < 0.001. (**c**) Regression graph representing the relation between methylation changes and nuts intake at 5 years. Dot lines on both sides of the solid line (linear regression line) represent 95% confidence band. MedDiet: Mediterranean diet.

**Figure 2:**
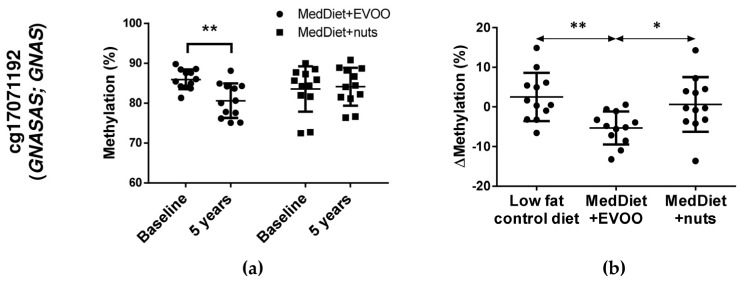
Representation of significant methylation changes of cg17071192. (**a**) Methylation mean and SD of each dietary group at baseline and at 5 years. Statistical analysis was performed by a Student-*t* test between 5 years and baseline for each diet. (**b**) Methylation changes (Mean and SD) for each participant and diet. Statistical analysis of differences in methylation changes among diets was performed by ANOVA (+Tukey’s multiple comparison test) or Kruskal-Wallis (+Mann-Whitney’s U), when appropriate. Significance is considered * *p* < 0.05, ** *p* < 0.01. EVOO: extra-virgin olive oil; MedDiet: Mediterranean diet.

After those preliminary analyses, CpGs were selected by B-values >0 and effect size ≤ −9 or ≥ 9 for both comparisons, as well as by showing non-significant differences in methylation values among the three dietary groups at baseline and showing significant differences throughout the intervention. Two CpGs were obtained: cg01081346 and cg17071192. The CpG cg01081346 presented an increase in methylation in MedDiet + nuts after five years of intervention (Figure 1a), and showed significant higher methylation changes in MedDiet + nuts subjects when comparing with the other two dietary groups (Figure 1b). In addition, nuts intake (g/day) at five years of follow-up was associated with an increase in cg01081346 methylation (Figure 1c). The other CpG (cg17071192) presented a methylation decrease in MedDiet + EVOO throughout the intervention (Figure 2a) and showed significant differences in methylation changes between the MedDiet + EVOO group and the other two groups (Figure 2b). The corresponding genes for these CpGs, according to the Illumina CG database, were *CPT1B/CHKB-CPT1B* (Carnitine palmitoyltransferase 1B/Choline kinase-like, Carnitine palmitoyltransferase 1B) for cg01081346, located at the TSS220, TSS1500 (*CPT1B*) and body of the gene (*CHKB-CPT1B*), and *GNAS/GNASAS* (Guanine Nucleotide Binding Protein (G Protein), Alpha Stimulating Activity Polypeptide 1/*GNAS* Antisense RNA 1) for cg17071192, located at the body of the gene (*GNASAS*) and TSS1500 (*GNAS*).

### 3.4. CpG cg01081346 Was Associated with PUFAs

**Figure 3:**
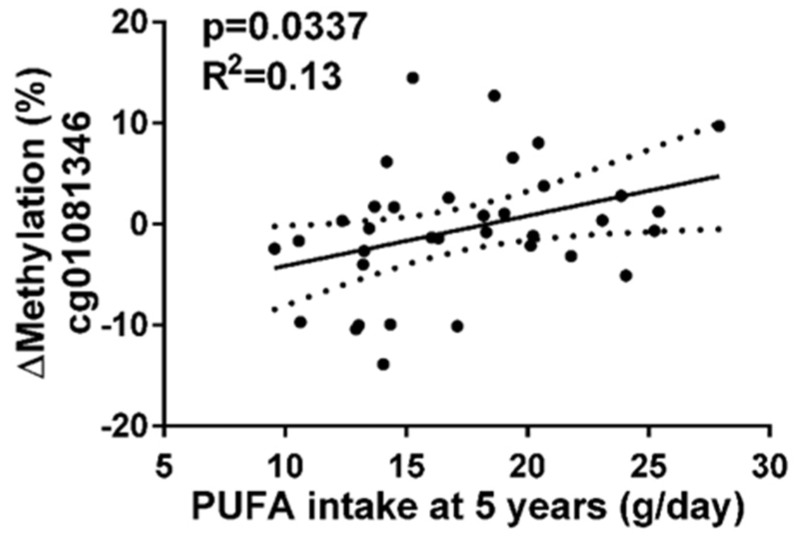
Regression graph between cg01081346 methylation and polyunsaturated fatty acids (PUFA) consumption at five years. Dot lines on both sides of the solid line (linear regression line) represent 95% confidence band.

In order to check whether fatty acids were related to methylation changes, other regression analyses were performed between methylation changes of CpGs and total lipids, saturated fatty acids, monounsaturated fatty acids (MUFAs), and polyunsaturated fatty acids (PUFAs) that were consumed at five years. Results showed that methylation of cg01081346 was associated with PUFAs (Figure 3), whereas cg17071192 presented no association.

## 4. Discussion

The current study demonstrates that a MedDiet pattern plus specific food supplements rich in different fat quality, in this case nuts and EVOO, could induce changes in methylation levels in specific CpGs of PWBCs. Therefore, further insights are added to previous reports where environmental factors, including the diet, were able to modify the epigenome [22].

MedDiets supplemented with nuts or EVOO have evidenced beneficial effects in individuals with cardiovascular risk [8]. Among these effects, decreased levels of LDL cholesterol and increased levels of HDL cholesterol were observed [23]. In the subset of participants selected for this study, there were differences among the three groups in HDL cholesterol. In fact, the low-fat dietary control group decreased HDL levels, whereas MedDiet + EVOO maintained the levels and MedDiet + nuts increased them. In most cases, there was a reduction in the incidence of diabetes, hypercholesterolemia and arterial pressure, indicating a general improvement in health status after each dietary intervention.

Regarding MedDiet supplements intake, although MedDiet + EVOO group presented differences in consumption of EVOO at five years and in changes between five years and baseline, MedDiet + nuts only showed differences in nuts intake at five years. This result could be influenced by the initial values of nuts consumption of the MedDiet + nuts group, which were higher than in the other two groups, although not statistically significant. Some dietary groups presented differences in other foods intake, probably due to dietary advice.

The IPA strategy revealed that the selected differentially methylated genes were mainly related to intermediate metabolism, inflammation, intracellular signals, and diabetes phenomena. These areas are interconnected and MedDiets supplemented with EVOO or nuts have been described as having a favourable impact in different features related to them, such as inflammatory parameters, hypertension, hyperlipidaemia, among others [8,9]. Thus, MedDiet, and especially the food supplements employed, EVOO and nuts, could be inducing changes in the DNA methylation, which in turn, may cause changes in the expression of some genes that are associated with these processes and related diseases. For instance, Rodríguez-Miguel et al. showed that an olive oil-enriched diet increased levels of global methylation in mammary glands and tumours [13]. In addition, adherence to MedDiet has been associated with changes in methylation in inflammatory-related genes in a previous study from this group [12]. Further studies of the relationship between significant genes and the pathways from IPA would be interesting to elucidate their putative involvement in MedDiet health beneficial effects.

After an exhaustive analytical selection in order to choose the most relevant CpGs that had diet-induced methylation changes, two CpGs were specifically analysed: cg01081346–*CPT1B/CHKB-CPT1B* and cg17071192-*GNAS/GNASAS*. Interestingly, three surrounding CpGs of 27 for *CPT1B/CHKB-CPT1B* and 13 CpGs of 129 for *GNAS/GNASAS* also showed significant differences (*p* < 0.05). These CpGs may be related to the expression of genes performing actions that were in agreement with the differentially methylated areas obtained in the IPA. For instance, *CPT1B* has been related to insulin sensitivity and cardiac risk [24,25]. This gene is implicated in the conversion of acyl-CoA and carnitine into acyl-carnitine in the outer membrane of mitochondria, determining the balance between glucose and fatty acid metabolism [26]. The levels of these molecules are very important in lymphocytes and mononuclear phagocytes metabolism [27]. On the other hand, *GNASAS* encodes for an antisense RNA transcript that regulates *GNAS*, which has been involved in glucose and energy regulation [28,29]. Although methylation levels were measured in PWBCs, which are not very related to some of the aforementioned functions, these cells could be proxies for less accessible tissues [30]. Indeed, differentially methylated CpGs in blood cells have been identified as mirroring the adipose tissue methylation pattern [31].

Nuts and EVOO are both rich in MUFAs and PUFAs [32,33]. In fact, in this study, methylation of CpG cg01081346 was associated with PUFA uptake. It is likely that this CpGs could be influenced by fat quality due to PUFAs consumed in nuts. Indeed, it has been described that the quality of dietary fat influences the methylation of genes [34]. Nevertheless, the CpG cg17071192 might change the methylation pattern due to other MedDiet nutrients or other compounds that are present in EVOO. For instance, polyphenols from EVOO have been described down-regulating proatherogenic genes [35], while some derivatives (hydroxityrosol, tyrosol, and their secoiridoid derivatives) have shown strong antioxidant and anti-inflammatory activity in vitro [36,37]. In addition, the stimulatory effect of phenolic extracts from EVOO and hydroxityrosol on cannabinoid receptor 1 (CB1) expression inversely correlated with DNA methylation at cannabinoid receptor 1 promoter and it was associated with reduced proliferation of human colon cancer cells [38].

The present investigation was not devoid of limitations. Firstly, the sample size is relatively low, but the results are plausible. Secondly, mRNA levels could be evaluated in order to determine EVOO and nuts roles on the modification of the methylation levels and in turn, the effect on the expression of genes with a function in the maintenance of a good health status.

## 5. Conclusions

Summing up, this study shows that an intervention with MedDiet + EVOO and MedDiet + nuts in the PREDIMED-Navarra trial are influencing the methylation of genes in PWBCs. This effect might be, in part, due to the fat profile of either EVOO or nuts. Specifically, methylation changes occurs in genes related to intermediate metabolism, diabetes, inflammation, and signal transduction process, such as *CPT1B* and *GNAS*, which are specifically involved in acyl-carnitine process in mitochondria, and glucose and energy regulation, respectively. Hence, the potential beneficial effects on health of Mediterranean dietary pattern supplemented with EVOO or nuts could be mediated, at least in part, through epigenetic mechanisms, where the quality of fat intake, such as PUFA consumption, is playing a mediating role.

## Figures and Tables

**Table 1:** Anthropometric, clinical and biochemical characteristics of the study population, with the statistical differences among the three dietary groups at baseline and after five-year follow-up.

	Low-Fat Control Diet (*n* = 12)		*p*^2^	MedDiet + EVOO (*n* = 12)		*p*^2^	MedDiet + nuts (*n* = 12)					
	Baseline	5 Years		Baseline	5 Years		Baseline	5 Years	*p*^2^	*p* 0^3^	*p* 5^3^	*p* Δ^3^
**Female, *n* (%)**	6 (50)			6 (50)			6 (50)			ns		
**Age(years) ^1^**	64.3 (3.9)	NA		63.5 (1.7)	NA		63.2 (2.1)	NA		ns		
**Weight(kg) ^1^**	73.0 (10.9)	73.1 (9.6)	ns	73.3 (9.4)	74.6 (10.7)	ns	71.6 (7.5)	73.2 (7.6)	ns	ns	ns	ns
**Waist circumference(cm) ^1^**	92.4 (9.6)	92.4 (9.5)	ns	90.1 (7.1)	93.8 (6.4)	ns	93.5 (6.3)	94.3 (6.7)	ns	ns	ns	ns
**BMI(kg/m^2^) ^1^**	27.3 (3.2)	27.4 (2.9)	ns	27.9 (1.5)	28.3 (1.8)	ns	28.1 (1.5)	28.8 (1.8)	ns	ns	ns	ns
**Glycemia(mg/dL) ^1^**	106.0 (27.6)	102.2 (18.0)	ns	129.5 (67.1)	120.8 (52.7)	ns	131.5 (57.4)	127.0 (34.9)	ns	ns	ns	ns
**HDL cholesterol(mg/dL) ^1^**	52.7 (14.5)	51.5 (13.3)	ns	55.2 (8.3)	55.1 (10.4)	ns	60.9 (9.1)	64.6 (12.0)	ns	ns	0.022	ns
**LDL cholesterol(mg/dL) ^1^**	132.0 (15.5)	112.3 (27.1)	ns	122.0 (30.5)	113.0 (26.3)	ns	119.1 (23.2)	123.1 (35.2)	ns	ns	ns	ns
**Total cholesterol(mg/dL) ^1^**	209.8 (21.0)	188.6 (27.7)	ns	203.4 (28.7)	191.6 (29.1)	ns	199.2 (24.3)	210.4 (41.2)	ns	ns	ns	ns
**Triglycerides(mg/dL) ^1^**	126.1 (49.6)	124.1 (40.7)	ns	131.2 (78.2)	117.6 (47.9)	ns	95.7 (24.6)	113.4 (40.0)	ns	ns	ns	ns
**Systolic arterialpressure (mmHg) ^1^**	148.9 (17.2)	150.1 (22.4)	ns	158.5 (19.1)	157.6 (20.5)	ns	142.9 (16.0)	147.7 (21.6)	ns	ns	ns	ns
**Diastolic arterialpressure (mmHg) ^1^**	85.7 (8.6)	85.8 (11.4)	ns	88.1 (8.4)	83.9 (10.0)	ns	87.9 (7.2)	87.2 (14.0)	ns	ns	ns	ns
**Diabetes, *n* (%)**	1 (8)	0 (0)	ns	4 (33)	0 (0)	0.046	6 (50)	0 (0)	0.014	ns	ns	0.022
**Hypercholesterolemia, *n* (%)**	8 (67)	0 (0)	0.005	9 (75)	1 (8)	0.005	10 (83)	1 (8)	0.003	ns	ns	ns
**Arterial hypertension, *n* (%)**	12 (100)	0 (0)	<0.001	11 (92)	1 (8)	0.004	10 (83)	4 (33)	ns	ns	ns	0.010
**Hypertriglyceridemia, *n* (%)**	1 (8)	2 (17)	ns	2 (17)	0 (0)	ns	0 (0)	0 (0)	ns	ns	ns	ns

^1^ Values are represented as Mean (SD).^2^ Values obtained by Student *t*-test, Wilcoxon signed-rank test, Sign test or Cochran test, as appropriate, comparing data from 5 years and baseline for each diet.^3^ Values obtained by chi-square test, Fisher’s exact test or Cochran test (for qualitative variables), and ANOVA test or Kruskal-Wallis test (for quantitative variables), as appropriate, from baseline data (*p* 0), from five years data (*p* 5) or from the difference between 5 years and baseline data (*p* Δ) among the three groups. *p* < 0.05 is considered significant. EVOO: extra-virgin olive oil; HDL: high-density lipoprotein; LDL: low-density lipoprotein; MedDiet: Mediterranean diet; NA: non-applicable; ns: non-significant.

**Table 2:** Energy and nutrient intake of participants and statistical differences among the three dietary groups at baseline and after five-year follow-up.

	Low-Fat Control Diet (*n* = 12)		*p*^2^	MedDiet + EVOO (*n* = 12)		*p*^2^	MedDiet + nuts (*n* = 12)		*p*^2^	*p* 0^3^	*p* 5^3^	*p* Δ^3^
Food/component (g/day)	Baseline	5 Years		Baseline	5 Years		Baseline	5 Years				
**Carbohydrates**	230.4 (46.7)	267.9 (60.1)	ns	239.4 (82.1)	248.7 (76.2)	ns	224.9 (52.3)	240.8 (44.9)	ns	ns	ns	ns
**Proteins**	89.9 (23.9)	96.8 (16.1)	ns	88.9 (16.0)	88.7 (14.8)	ns	91.5 (16.0)	95.0 (18.9)	ns	ns	ns	ns
**Fat**	100.5 (24.2)	97.2 (19.5)	ns	108.2 (30.3)	117.3 (25.6)	ns	108.9 (19.4)	124.1 (25.1)	ns	ns	0.017	ns
**MUFA**	51.8 (11.8)	51.3 (11.0)	ns	56.6 (16.4)	63.9 (10.6)	ns	55.3 (12.4)	64.6 (13.0)	ns	ns	0.014	ns
**SFA**	23.3 (7.2)	22.5 (7.3)	ns	26.7 (8.9)	25.0 (6.8)	ns	24.8 (5.3)	28.8 (8.1)	ns	ns	ns	ns
**PU FA**	15.8 (5.5)	15.5 (5.0)	ns	16.8 (6.3)	16.9 (4.2)	ns	18.6 (4.7)	19.7 (4.1)	ns	ns	ns	ns
**Vegetables**	302.1 (90.2)	352.2 (104.2)	ns	302.3 (117.8)	370.2 (58.2)	ns	298.9 (71.1)	372.7 (129.1)	ns	ns	ns	ns
**Fish**	73.5 (32.6)	93.6 (37.5)	ns	90.2 (33.8)	82.6 (25.7)	ns	91.2 (33.5)	100.4 (39.9)	ns	ns	ns	ns
**Fruits**	291.6 (145.1)	431.4 (237.1)	0.009	319.8 (181.9)	459.3 (109.9)	ns	367.2 (163.2)	500.8 (186.7)	0.034	ns	ns	ns
**Legumes**	17.9 (9.9)	14.7 (6.4)	ns	16.0 (4.7)	19.6 (7.5)	ns	15.5 (3.5)	20.4 (5.8)	0.019	ns	ns	0.034
**Nuts**	12.2 (18.2)	5.5 (8.8)	ns	9.9 (16.4)	11.3 (8.3)	ns	20.5 (15.1)	28.2 (14.2)	ns	ns	<0.001	ns
**EVOO**	29.6 (26.3)	32.9 (26.2)	ns	39.6 (24.7)	66.4 (8.1)	0.003	25.0 (30.0)	33.8 (32.4)	ns	ns	0.002	0.049
**Rest of olive oils**	16.7 (24.6)	16.7 (24.6)	ns	10.0 (15.8)	0 (0)	0.049	26.3 (27.6)	25.8 (32.6)	ns	ns	ns	ns
**Red meat**	63.2 (42.5)	59.8 (38.1)	ns	71.7 (42.3)	39.5 (28.0)	0.045	57.5 (29.8)	54.8 (32.6)	ns	ns	ns	ns
**White meat**	54.9 (28.6)	63.0 (25.4)	ns	39.5 (22.4)	36.7 (26.8)	ns	43.1 (23.2)	47.7 (26.4)	ns	ns	ns	ns
**Pastries**	35.3 (18.2)	36.9 (27.3)	ns	43.4 (24.5)	29.8 (21.4)	ns	23.9 (21.1)	37.6 (27.6)	ns	ns	ns	ns
**Total energy (kcal/day)**	2310 (420)	2412 (475)	ns	2378 (651)	2470 (513)	ns	2349 (432)	2535 (452)	ns	ns	ns	ns

Values are represented as Mean (SD).^1^ Values obtained by Student *t*-test, Wilcoxon signed-rank test or Sign test, as appropriate, comparing data from 5 years and baseline for each diet.^2^ Values obtained by ANOVA test or Kruskal-Wallis test, as appropriate, from baseline data (*p* 0), from 5 years data (*p* 5) or from the difference between 5 years and baseline data (*p* Δ) among the three groups. *p* < 0.05 is considered significant. EVOO: extra-virgin olive oil; MedDiet: Mediterranean diet; ns: non-significant; MUFA: monounsaturated fatty acids; PUFA: polyunsaturated fatty acids; SFA: saturated fatty acids.

## Supplementary Materials

The following are available online at http://www.mdpi.com/2072-6643/10/1/15/s1, Table S1: Pearson correlation values of differences in types of leukocytes and consumption at 5 years of extra-virgin olive oil (EVOO) and nuts, and methylation changes of cg01081343 and cg17071192, Table S2: Differences among dietary groups in the composition of blood cells, Table S3: Canonical pathways (Ingenuity Pathway Analysis) associated with differentially methylated CpGs selected from MedDiet + EVOO vs. low-fat control diet LIMMA analysis, Table S4: Canonical pathways (Ingenuity Pathway Analysis) associated with differentially methylated CpGs selected from MedDiet + nuts vs. low-fat control diet LIMMA analysis.
