# Supplementary material for: Impact of Consuming Extra-Virgin Olive Oil or Nuts within a Mediterranean Diet on DNA Methylation in Peripheral White Blood Cells within the PREDIMED-Navarra Randomized Controlled Trial: A Role for Dietary Lipids
Source: Nutrients. 2017 Dec 23;10(1):15. doi: 10.3390/nu10010015 (PMC5793243; doi:10.3390/nu10010015)
Supplement: Supplementary file 1 [file nutrients-10-00015-s001.pdf]

Table S1. Pearson correlation values of differences in types of leukocytes and consumption at 5 years of extra-virgin olive oil (EVOO) and nuts, and methylation changes of cg01081343 and cg17071192.

Correlation	r	p-value
**cg01081343 vs.**		
Leukocytes	0.042	0.842
Lymphocytes	0.048	0.821
Monocytes	0.250	0.228
Neutrophils	-0.134	0.522
Eosinophils	0.060	0.777
Basophils	0.096	0.649
**cg17071192 vs.**		
Leukocytes	0.245	0.237
Lymphocytes	-0.289	0.161
Monocytes	0.196	0.348
Neutrophils	0.184	0.378
Eosinophils	0.148	0.481
Basophils	-0.016	0.939
**Nuts vs.**		
Leukocytes	0.386	0.062
Lymphocytes	0.023	0.916
Monocytes	0.302	0.152
Neutrophils	-0.078	0.719
Eosinophils	-0.115	0.593
Basophils	0.012	0.955
**Extra-virgin olive oil vs.**		
Leukocytes	-0.097	0.653
Lymphocytes	-0.103	0.634
Monocytes	-0.235	0.268
Neutrophils	-0.227	0.286
Eosinophils	-0.249	0.242
Basophils	0.003	0.989

Correlation values were obtained after Pearson correlations.
$p<0.05$ is considered significant.

Table S2. Differences among dietary groups in the composition of blood cells.

	p-value ANOVA	p-value Low-fat vs. MedDiet+EVOO	p-value Low-fat vs. MedDiet+nuts	p-value MedDiet+EVOO vs. MedDiet+nuts
Leukocytes	0.118			
Lymphocytes	0.065			
Monocytes	0.963			
Neutrophils	0.049	0.077	0.997	0.089
Eosinophils	0.290			
Basophils	0.354			

Statistical analysis was performed using ANOVA (+ Tukey's multiple comparison test). $p<0.05$ is considered significant.

Table S3. Canonical pathways (Ingenuity Pathway Analysis) associated with differentially methylated CpGs selected from MedDiet+EVOO vs. low-fat control diet LIMMA analysis.

Ingenuity Canonical Pathways	-log(p-value)	Ratio	Genes
Hepatic Fibrosis / Hepatic Stellate Cell Activation	2,76E00	3,83E-02	TLR4,COL19A1,PROK1,IFNGR2,COL20A1,COL11A2,TNFRSF1B
Fatty Acid Activation	2,20E00	1,54E-01	SLC27A5,ACSBG2
Crosstalk between Dendritic Cells and Natural Killer Cells	2,01E00	4,49E-02	TLR4,FSCN2,TNFRSF1B,CAMK2B
Aryl Hydrocarbon Receptor Signaling	1,99E00	3,57E-02	AHRR,NFIC,POLA1,ALDH1A2,ALDH3B2
γ-linolenate Biosynthesis II (Animals)	1,97E00	1,18E-01	SLC27A5,ACSBG2
Mitochondrial L-carnitine Shuttle Pathway	1,97E00	1,18E-01	SLC27A5,ACSBG2
LPS/IL-1 Mediated Inhibition of RXR Function	1,73E00	2,70E-02	TLR4,SLC27A5,ALDH1A2,ACSBG2,TNFRSF1B,ALDH3B2
Fatty Acid β-oxidation I	1,44E00	6,25E-02	SLC27A5,ACSBG2
Uracil Degradation II (Reductive)	1,43E00	2,50E-01	DPYD
Thymine Degradation	1,43E00	2,50E-01	DPYD
Leukocyte Extravasation Signaling	1,32E00	2,38E-02	MMP28,RAP1GAP,CLDN16,DLC1,CLDN22

Ratio represents differentially methylated genes/genes in the pathway. A –log(p-value)>1.301 is considered significant, which corresponds to a $p<0.05$. CpG: CG site; EVOO: extra-virgin olive oil; LIMMA: Linear Models for Microarray Data; MedDiet: Mediterranean diet

Table S4. Canonical pathways (Ingenuity Pathway Analysis) associated with differentially methylated CpGs selected from MedDiet+nuts vs. low-fat control diet LIMMA analysis.

Ingenuity Canonical Pathways	-log(p-value)	Ratio	Genes
Axonal Guidance Signaling	2,78E00	3,56E-02	PIK3C2B,FYN,PLXNC1,GNAS,SEMA5A,EPHA4,PLCH2,PRKCZ,NFATC1, WNT10A,SRGAP1,PRKAG2,PLXNB2,ABLIM2,LNPEP,SHANK2
Sperm Motility	2,49E00	5,60E-02	NPPB,PDE2A,GNAS,CACNA1H,PRKAG2,PLCH2,PRKCZ
Sumoylation Pathway	2,44E00	6,25E-02	TP53,ETS1,DAXX,AR,CEBPA,MAP3K5
Role of p14/p19ARF in Tumor Suppression	2,38E00	9,30E-02	TP53,RB1,PIK3C2B,TTF1
Myc Mediated Apoptosis Signaling	2,35E00	7,14E-02	TP53,PIK3C2B,IGF1R,PRKCZ,BCL2
Adipogenesis pathway	2,33E00	5,22E-02	TP53,HDAC9,ZNF423,NR1D2,EBF1,HDAC4,CEBPA
SAPK/JNK Signaling	2,27E00	5,77E-02	TP53,PIK3C2B,DAXX,DUSP10,MAP3K5,NFATC1
Molecular Mechanisms of Cancer	2,27E00	3,48E-02	TP53,RB1,FYN,DAXX,SYNGAP1,PIK3C2B,GNAS,WNT10A,PRKAG2, RBPJ,MAP3K5,PRKCZ,BCL2
p53 Signaling	2,13E00	5,41E-02	TP53,HDAC9,RB1,PIK3C2B,COQ8A,BCL2
Telomerase Signaling	2,13E00	5,41E-02	TP53,ETS1,HDAC9,RB1,PIK3C2B,HDAC4
Growth Hormone Signaling	2,09E00	6,17E-02	PIK3C2B,CEBPA,IGF1R,RPS6KA2,PRKCZ
Neuropathic Pain Signaling In Dorsal Horn Neurons	2,08E00	5,26E-02	PIK3C2B,GRM8,GRIA1,PRKAG2,PLCH2,PRKCZ
Unfolded protein response	2,03E00	7,41E-02	PDIA2,CEBPA,MAP3K5,BCL2
Role of NFAT in Cardiac Hypertrophy	2,01E00	4,17E-02	HDAC9,PIK3C2B,HDAC4,GNAS,PRKAG2,IGF1R,PLCH2,PRKCZ
Synaptic Long Term Potentiation	1,98E00	5,00E-02	GRM8,GRIA1,PRKAG2,PPP1R14A,PLCH2,PRKCZ
Induction of Apoptosis by HIV1	1,87E00	6,67E-02	TP53,DAXX,MAP3K5,BCL2
Prostate Cancer Signaling	1,83E00	5,32E-02	TP53,RB1,PIK3C2B,AR,BCL2
Melanocyte Development and Pigmentation Signaling	1,81E00	5,26E-02	PIK3C2B,GNAS,PRKAG2,RPS6KA2,BCL2
Cell Cycle: G1/S Checkpoint Regulation	1,77E00	6,25E-02	TP53,HDAC9,RB1,HDAC4
Protein Kinase A Signaling	1,73E00	3,06E-02	PDE2A,GNAS,PPP1R1B,DUSP10,MYLK2,PTPRS,PRKAG2,PPP1R14A, PLCH2,NFATC1,PRKCZ,DUSP16
Notch Signaling	1,69E00	7,89E-02	NOTCH4,RBPJ,DLL3
Aryl Hydrocarbon Receptor Signaling	1,67E00	4,29E-02	TP53,AHRR,RB1,RARA,POLA1,ALDH3A1
Netrin Signaling	1,66E00	7,69E-02	PRKAG2,ABLIM2,NFATC1
Chronic Myeloid Leukemia Signaling	1,66E00	4,81E-02	TP53,HDAC9,RB1,PIK3C2B,HDAC4
Hepatic Fibrosis / Hepatic Stellate Cell Activation	1,64E00	3,83E-02	COL19A1,COL23A1,COL9A3,IGF1R,COL18A1,COL11A2,BCL2
CREB Signaling in Neurons	1,62E00	3,80E-02	PIK3C2B,GNAS,GRM8,GRIA1,PRKAG2,PLCH2,PRKCZ
Ovarian Cancer Signaling	1,62E00	4,17E-02	TP53,RB1,PIK3C2B,WNT10A,PRKAG2,BCL2
Synaptic Long Term Depression	1,60E00	4,11E-02	GNAS,GRM8,GRIA1,IGF1R,PLCH2,PRKCZ
Glioma Signaling	1,56E00	4,55E-02	TP53,RB1,PIK3C2B,IGF1R,PRKCZ
Mitochondrial L-carnitine Shuttle Pathway	1,56E00	1,18E-01	SLC27A2,CPT1B
Superpathway of Inositol Phosphate Compounds	1,55E00	3,42E-02	PIK3C2B,FYN,PHOSPHO1,PPP1R1B,DUSP10,PPP1R14A,PLCH2,DUSP16
β-alanine Degradation I	1,52E00	5,00E-01	ABAT
Valine Degradation I	1,52E00	1,11E-01	ABAT,HIBADH
Cyclins and Cell Cycle Regulation	1,49E00	5,13E-02	TP53,HDAC9,RB1,HDAC4
Role of Oct4 in Mammalian Embryonic Stem Cell Pluripotency	1,48E00	6,52E-02	TP53,RB1,RARA
DNA damage-induced 14-3-3σ Signaling	1,47E00	1,05E-01	TP53,HUS1
3-phosphoinositide Biosynthesis	1,46E00	3,52E-02	PIK3C2B,FYN,PHOSPHO1,PPP1R1B,DUSP10,PPP1R14A,DUSP16
NGF Signaling	1,46E00	4,27E-02	TP53,PIK3C2B,RPS6KA2,MAP3K5,PRKCZ
p38 MAPK Signaling	1,46E00	4,27E-02	TP53,DAXX,DUSP10,RPS6KA2,MAP3K5
Glioblastoma Multiforme Signaling	1,44E00	3,77E-02	TP53,RB1,PIK3C2B,WNT10A,IGF1R,PLCH2
D-myo-inositol-5-phosphate Metabolism	1,42E00	3,73E-02	PHOSPHO1,PPP1R1B,DUSP10,PPP1R14A,PLCH2,DUSP16
Dopamine-DARPP32 Feedback in cAMP Signaling	1,41E00	3,70E-02	GNAS,PPP1R1B,PRKAG2,PPP1R14A,PLCH2,PRKCZ
Role of Tissue Factor in Cancer	1,40E00	4,10E-02	TP53,PDIA2,PIK3C2B,FYN,RPS6KA2
Small Cell Lung Cancer Signaling	1,39E00	4,76E-02	TP53,RB1,PIK3C2B,BCL2
GPCR-Mediated Nutrient Sensing in Enteroendocrine Cells	1,38E00	4,71E-02	GNAS,PRKAG2,PLCH2,PRKCZ
Fatty Acid α-oxidation	1,35E00	9,09E-02	ALOX12B,ALDH3A1
Thyroid Hormone Biosynthesis	1,34E00	3,33E-01	TPO
4-aminobutyrate Degradation I	1,34E00	3,33E-01	ABAT
Docosahexaenoic Acid (DHA) Signaling	1,34E00	5,77E-02	PIK3C2B,BIK,BCL2
Type II Diabetes Mellitus Signaling	1,33E00	3,94E-02	PIK3C2B,SLC27A2,PRKAG2,MAP3K5,PRKCZ
HER-2 Signaling in Breast Cancer	1,33E00	4,55E-02	TP53,PIK3C2B,MAP3K5,PRKCZ
Gap Junction Signaling	1,32E00	3,53E-02	PIK3C2B,DBN1,GNAS,PRKAG2,PLCH2,PRKCZ

Ratio represents differentially methylated genes/genes in the pathway. A –log(p-value)>1.301 is considered significant, which corresponds to a p<0.05. CpG: CG site; LIMMA: Linear Models for Microarray Data; MedDiet: Mediterranean diet
